# Validated HPLC–PDA methodology utilized for simultaneous determination of Etoricoxib and Paracetamol in the presence of Paracetamol toxic impurities

**DOI:** 10.1186/s13065-022-00904-z

**Published:** 2022-12-02

**Authors:** Mona A. Abdel Rahman, Mohamed R. Elghobashy, Hala E. Zaazaa, Shimaa A. Atty, Sally S. El-Mosallamy

**Affiliations:** 1 Analytical Chemistry Department, Faculty of Pharmacy, October 6 University, 6 October City, PO Box 12858, Giza, Egypt; 2 Analytical Chemistry Department, Faculty of Pharmacy, Cairo University, Kasr El-Aini St, Cairo, 11562 PO Egypt; 3 Pharmaceutical Chemistry Department, Egyptian Drug Authority, 51 Wezaret El-Zeraa St, Cairo, Egypt

**Keywords:** 4-Aminophenol, Para-hydroxy acetophenone, HPLC–PDA, Paracetamol, Etoricoxib, Toxic impurities

## Abstract

Etoricoxib (ETO), Paracetamol (PCM), and two toxic impurities for Paracetamol impurity K (4-aminophenol (PAP)) and impurity E (para-hydroxy acetophenone (PHA)) were separated using a simple and selective HPLC method that was tested for the first time. PCM is a commonly used analgesic and antipyretic medication that has recently been incorporated into COVID-19 supportive treatment. Pharmaceuticals containing PCM in combination with other analgesic-antipyretic drugs like ETO help to improve patient compliance. The studied drugs and impurities were separated on a GL Sciences Inertsil ODS-3 (250 × 4.6) mm, 5.0 µm column, and linear gradient elution was performed using 50 mM potassium dihydrogen phosphate adjusted to pH 4.0 with ortho-phosphoric acid and acetonitrile as mobile phase at 2.0 mL/min flow rate at 25 °C and UV detection at 220 nm. The linearity range was 1.5–30.0 µg/mL for ETO and PCM while 0.5–10.0 µg/mL for PAP and PHA, with correlation coefficients (r) for ETO, PCM, PAP, and PHA of 0.9999, 0.9993, 0.9996, and 0.9998, respectively. The proposed method could be used well for routine analysis in quality control laboratory.

## Introduction

The global spread of COVID-19 has resulted in an unprecedented disaster. In the early stages, the most common symptom is fever due to the onset of a COVID-19-mediated cytokine storm. NSAIDs are among the most extensively used medicines owing to their efficacy in reducing pain and inflammation and their inclusion in the WHO's Model List of Essential Medicine [1].

Paracetamol (PCM) and Etoricoxib (ETO) are anti-inflammatory, analgesic-antipyretic drugs [2] and approved to treat COVID symptoms. PCM is an N-(4-hydroxyphenyl) acetamide [3]. PCM is a popular over-the-counter analgesic for mild to moderate pain, such as headaches, musculoskeletal discomfort, and fever [4], and has recently been used in the adjunctive treatment of COVID-19 as it acts as an antipyretic for fever resulting from infection with COVID-19 [5–7].

**Fig. 1:**
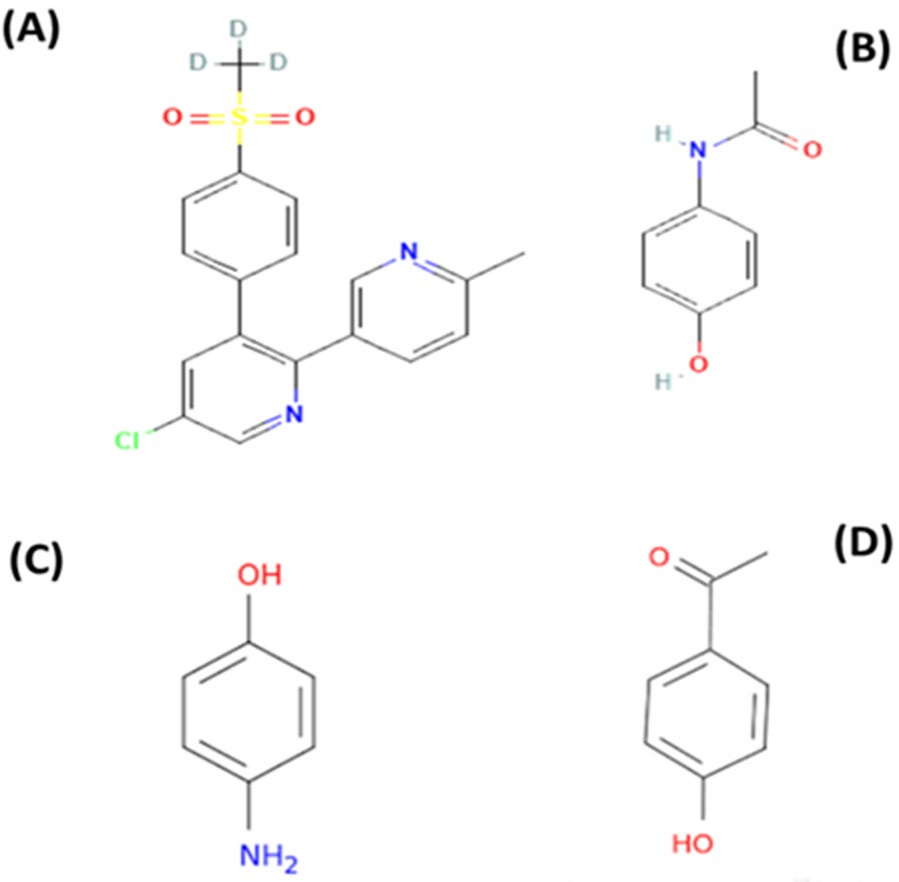
Structure of **a** Etoricoxib, **b** Paracetamol, **c** 4-aminophenol and **d** Para-hydroxy acetophenone

ETO is 5 chloro-6’- methyl-3- [-4- (methyl sulfonyl) phenyl]-2,3’- bipyridine. It is a COX-2-selective NSAID drug with a better COX-1 to COX-2 selectivity ratio than other COX-2-selective NSAIDs [8]. It is also used to treat osteoarthritis, rheumatoid arthritis, gouty arthritis, chronic low back pain, and acute pain [9], and to treat COVID-19 by suppressing cytokine storm [10]. Figure 1A and B show the structures of PCM and ETO.

During the manufacture of Active Pharmaceutical Ingredients (APIs), some of the starting materials, intermediates, reagents, and reaction by-products end up in the final products as impurities [11].

The presence of impurity in a drug product is mostly a quality issue, as it may compromise the drug's efficacy and may cause potential adverse effects on patients [12].

Each impurity found in a pharmaceutical product must be extensively inspected, both qualitatively and quantitatively, and undergo toxicological testing if necessary [13]. Accordingly, it is necessary to develop a technique for determining pharmaceuticals as well as their impurities at minute levels that may be harmful and have toxic effects.

4-aminophenol (impurity K) (Para aminophenol) is the most common co-existing impurity of PCM in medicinal formulations arising from either degradation or synthesis [14]. 1-(4-hydroxyphenyl) ethanone (impurity E) (para-hydroxy acetophenone) is an impurity of PCM, according to British pharmacopeia [15]. Figure 1C and D show the structures of PAP and PHA.

Several methods, like HPLC [16–26], HPTLC [27], spectrophotometric [28–31], chemmometric method [32–34], and electrochemical methods [35–38], have been reported for the assay of PCM. Several methods have been reported for the assay of ETO, including HPLC methods [39–44], HPTLC [45], spectrophotometric method [46], LC–MS/MS method [47], and ion-selective electrode method [48].

Both PCM and ETO were determined by several reported methods, such as HPLC [49–53], HPTLC [54], and spectrophotometric methods [55]. The published methods disregarded the possible impact of PCM impurities such as PHA (hepatotoxic) and PAP (nephrotoxic and teratogenic toxicity), despite the toxicity of these impurities. Also, according to the literature, there is no chromatographic method for determination of PCM and ETO in the presence of PCM impurities.

This work aims to develop, optimize, and validate a simple, sensitive, and selective RP-HPLC technique to be the first method for the simultaneous determination of PCM, ETO, and PCM potential impurities in bulk material and their pharmaceutical formulation.

## Experimental

### Instrumentation

HPLC SHIMADZU/LC-2030C 3D Plus with vacuum degasser and column GL Sciences Inertsil ODS-3 (250 × 4.6) mm, 5.0 µm. A photodiode Array Detector (PDA), a quaternary pump, a 100 µL (μL) loop, as well as an autosampler injector were employed. The LabSolutions software (v5.90/2017, Shimadzu Corporation, Kyoto, Japan) was used to record and evaluate the data. An electronic balance (Vibra, Japan), a 0.45 µm nylon membrane filter (Millipore, Ireland), an ultrasonic power sonicator (Model 410), and a pH meter (Jenway 3510, UK) were used.

### Chemicals and reagents

#### Pure standard

Pure standard ETO and PCM were donated by SIGMA Pharmaceutical Industries, Cairo, Egypt, and according to the company's analytical certificate, the purity of ETO and PCM was 99.5% and 99.94%, respectively. PAP and PHA were purchased from Sigma-Aldrich, and the purity of PAP and PHA was certified to be 99.73% and 99.61%, respectively.

#### Reagents

All chemicals and solvents employed in this experiment were of analytical grade. HPLC-grade acetonitrile and methanol were purchased from Sigma-Aldrich, Belgium. Potassium dihydrogen phosphate was supplied by El-NASR Pharmaceutical Chemical Co. (Abu-Zabaal, Cairo, Egypt). Double-distilled water (Otsuka Pharmaceutical Co., Cairo, Egypt) was used. Phosphate buffer (pH = 4) was prepared by dissolving about 6.8 gm of potassium dihydrogen phosphate in 1000 mL of doubled distilled water; the pH was adjusted using orthophosphoric acid (Sigma-Aldrich, Switzerland).

#### Pharmaceutical dosage form

Intacoxia-P^®^ tablet (Batch no: 5/UA/2017) was purchased from Aagya Biotech Pvt Ltd (Manglaur Roorkee, Uttarakhand, India). Intacoxia-P^®^ tablet is labelled to contain ETO 60 mg and 325 mg of PCM per tablet.

### Stock and working standard solutions

In four separate 25 mL volumetric flasks, 25 mg of ETO, PCM, PAP, and PHA were accurately weighed before being dissolved in 15 mL of methanol and sonicated for 10 min. The volume was raised to the mark with methanol, yielding a final concentration of 1.0 mg/mL. Then, from their standard stock solutions, 5, 5, 1.3, and 1.3 mL of ETO, PCM, PAP, and PHA, respectively, were transferred into four separate 50 mL volumetric flasks and the volume was raised to the mark with methanol to obtain standard working solutions of 100, 100, 26, and 26 µg/mL for ETO, PCM, PAP, and PHA, respectively.

### Procedure

#### Chromatographic conditions

The components were separated using a GL Sciences Inertsil ODS-3 column (250 × 4.6) mm, 5.0 µm with a linear gradient elution of 50 mM potassium dihydrogen phosphate adjusted to pH 4.0 with ortho-phosphoric acid (solvent A) and acetonitrile (solvent B). The applied gradient program is shown in Table 1, solvents were filtered through a 0.45 µm Millipore membrane filter and degassed ultrasonically for 15 min before being injected into the HPLC system at a flow rate of 2.0 mL/min. The PDA detector's wavelength was 220 nm, and all chromatographic separations were performed at room temperature (25 ± 2 °C).

**Table 1:** Gradient program of the RP-HPLC method for separation of the studied components

Time, min	Phosphate buffer, %	Acetonitrile, %
0 ^a^	93	7
0–2.5	75	25
2.5–5	50	50
5–7.5	25	75
7.5–9.5	93	7

^a^ gradient profile starting with 93: 7 (Phosphate buffer: Acetonitrile)

#### Construction of calibration graphs

Into 10 mL volumetric flasks, aliquots of standard working solution were diluted with the mobile phase (50:50 v/v, phosphate buffer: acetonitrile) to achieve concentrations ranging from 1.5 to 30 μg/mL for ETO and PCM and 0.5‒10 μg/mL for PAP and PHA. Diluted standard solutions of varying concentrations were subsequently injected by auto-sampler (80 µL volume) in triplicates into the HPLC system and chromatographed under the above-mentioned chromatographic conditions. Using a PDA detector, chromatographic peaks were obtained at 220 nm.

#### Analysis of tablet formulation

Ten tablets were weighed to determine the mean weight, then they were finely ground. The weight of the crushed powder corresponding to 18.5 mg of ETO and 100 mg of PCM was accurately weighed out and dissolved in 50 mL of the mobile phase (50:50 v/v, phosphate buffer: acetonitrile) in a 100 mL volumetric flask. After 15 min of sonication, the volume was completed to the mark with the same solvent. After that, the solution was filtered via a 0.45 µm membrane filter, yielding an initial stock solution claimed to contain 0.18 mg/mL of ETO and 1.0 mg/mL of PCM. The obtained solution was further diluted using the mobile phase to reach the final concentration 1.8 µg/mL and 10 µg/mL for ETO and PCM respectively, then injected in triplicate. The separation was accomplished using the chromatographic conditions described above. The concentrations of the listed drugs were determined using the calculated regression equations. We applied a standard addition technique to further evaluate the suggested method's accuracy.

### Method validation

The proposed method was validated in accordance to ICH guidelines [56].

## Results and discussion

The current work intends to develop and validate the first chromatographic method for determining PCM and ETO simultaneously as well as PCM toxic impurities.

### Method development and optimization

The chromatographic settings were optimized to produce a good resolution of the investigated components with sharp symmetric peaks in a short run time.

**Fig. 2:**
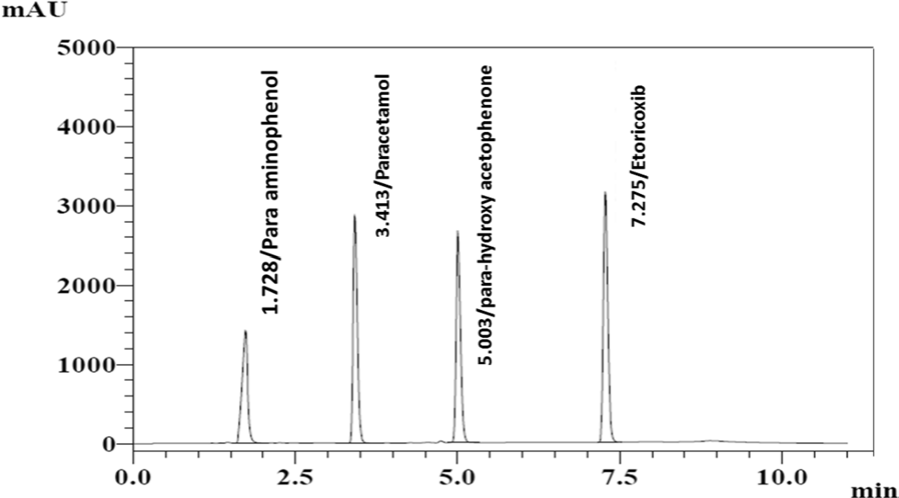
HPLC chromatogram of 4-aminophenol (10 μg/mL, t_R_: 1.728 min), paracetamol (30 μg/mL, t_R_: 3.413 min), para-hydroxy acetophenone (10 μg/mL, t_R_: 5.003 min), and Etoricoxib (30 μg/mL, t_R_: 7.275 min) using a linear gradient elution of solvent A (50 mM of potassium dihydrogen phosphate, adjusted to pH 4.0 with ortho-phosphoric acid) and solvent B (acetonitrile) as a mobile phase at a flow rate of 2.0 mL/min at 220 nm

Following an examination of different solvent compositions, methanol was chosen for preparing the stock and working standard solutions since all standards displayed acceptable solubility at the examined concentrations. Components of interest were separated using the suggested HPLC gradient elution method combined with UV detection at 220 nm. Figure 2 shows typical chromatograms of well-defined symmetrical peaks for PAP, PCM, PHA, and ETO mixtures.

Various experimental factors which affect separation were studied, including:

#### Choosing a suitable wavelength

We adjusted the PDA detector at several wavelengths to find the optimal one regarding the sensitivity and peak shape of the components under study, and all compounds had reasonable UV absorption at 220 nm. As a result, the 220 nm wavelength was selected for the investigation and quantification of ETO, PCM, and impurities of PCM.

#### Selection of the column

The HPLC column was chosen after trying several packing materials. C8, and C18 columns such as GL Sciences Inertsil ODS-3 column (250 × 4.6) mm, 5.0 µm and Kinetex C_8_ (4.6 × 100 mm, 5 µm; Phenomenex, USA), were tried. Employing a C8 column, some of the components (polar components like PAP and PCM) were retained in the column with a long separation time, which was most likely due to the high polarity of the C8 column. The separation was enhanced and the best results with excellent sharp peaks were obtained by utilizing a GL Sciences Inertsil ODS-3 column (250 × 4.6) mm, 5.0 µm instead of C8 columns. Furthermore, the influence of column temperatures ranging from 25 to 40 °C was examined. However, no enhancement in terms of analysis time was found upon increasing column temperature due to the low viscosity of the mobile phase. As a result, no significant variations in retention times were seen over the temperature range investigated. Finally, the temperature was kept at 25 °C.

#### Selection of mobile phase

The examined substances show a significant difference in lipophilicity (log P) of 0.47, 0.51, 1.23, and 2.79 for PAP, PCM, PHA, and ETO, respectively.

At the beginning of the study, isocratic elution was used to separate the four components using varied ratios of water/methanol, water/ethanol, and water/acetonitrile as mobile phases.

In these trials, there was either inadequate separation or overlapping peaks, particularly PCM and PAP peaks, as their polarity is nearly the same, while ETO took more time to be separated. So, we shifted to gradient elution with the same mobile phases, acetonitrile was more suitable for the separation of the studied components than methanol and ethanol, where acetonitrile is less polar than methanol and ethanol. In addition, the UV cut-off of acetonitrile (190 nm) is less than methanol (210 nm) and ethanol (210 nm).

Buffer was tried instead of water and pH was varied from 2 to 8, using phosphate buffer, pH 4.0 resulted in an obvious improvement giving sharp peak shapes and excellent separation of the mixture simultaneously with symmetric peaks in short run time.

Finally, a linear gradient elution using 50 mM potassium dihydrogen phosphate adjusted to pH 4.0 with ortho-phosphoric acid (solvent A) and acetonitrile (solvent B) was conducted as described in Table 1.

The optimal resolution, unambiguous baseline separation with adequate retention times, and symmetric peaks of the investigated drugs were obtained. UV detection was carried out at 220 nm. A good resolution was achieved with retention times at t_R_ of 1.728 ± 0.01, 3.413 ± 0.01, 5.003 ± 0.01, 7.275 ± 0.01 min for PAP, PCM, PHA, and ETO, respectively, as shown in Fig. 2.

#### Effect of flow rate

Different flow rates of the mobile phase were tried, ranging from 1.0 to 2.0 mL/min, to separate the analytes’ peaks from the mobile phase. The best flow rate for the effective elution of the drugs was 2.0 mL/min, giving better peak shape and a shorter retention time for all analytes while keeping acceptable peak resolution.

#### System suitability testing

To assess the performance of the operating system for the required separation, Table 2 shows the results of system suitability parameters, and satisfactory results were obtained according to USP pharmacopoeia [57], demonstrating perfect baseline separation of the separated peaks and high selectivity of the suggested method.

**Table 2:** Parameters required for the proposed HPLC method's system suitability testing

Parameters	PAP	PCM	PHA	ETO	Reference value (50)
Resolution (R_s_)^a^	12.290	14.498		20.296	R_s_ > 2
Selectivity factor (α)^b^	2.372	1.545		1.504	α > 1
Capacity factor (K)	2.456	5.827	7.005	8.549	1 < K < 10
Number of theoretical plates (N)	2437	9838	19595	41923	N > 2000
Tailing factor (T)	1.550	1.352	1.352	1.280	T ≤ 2

^a^ Resolution (R_s_) = 2(t_RB_—t_RA_) / (W_B+_W_A_), where *t*_*R*_ is the retention time and w is the peak width calculated for each of the two successive peaks

^b^ Selectivity (α) = *k’*_2_/*k’*_1_ calculated for each of two successive peaks

#### Robustness

The robustness of the HPLC technique was evaluated by analyzing the influence of slight modifications on the chromatographic conditions, such as the percentage of buffer ± 1% in the mobile phase components, the flow rate of the mobile phase (2.0 ± 0.1 mL/min), and pH (4 ± 0.2). Even minor changes to the perfect conditions had no discernible effect on retention times, tailing factor, and resolution of the examined components, which was proved by the low %RSD values, indicating the reliability of the proposed method during routine use (Table 3).

**Table 3:** Parameters related to robustness evaluation

Drugs	Variable parameters	Degree of variation	Retention time	Tailing factor	Resolution
ETO	Mobile phase ratio	Buffer 92%	7.214	1.234	20.306
		Buffer 93%	7.275	1.280	20.296
		Buffer 94%	7.232	1.252	20.212
		Mean ± SD	7.240 ± 0.031	1.255 ± 0.023	20.271 ± 0.051
		%RSD	0.034	1.846	0.254
	Flow Rate	1.9	7.243	1.269	20.242
		2.0	7.275	1.280	20.296
		2.1	7.253	1.250	20.215
		Mean ± SD	7.257 ± 0.016	1.266 ± 0.015	20.251 ± 0.033
		%RSD	0.225	1.198	0.166
	pH	3.8	7.253	1.278	20.272
		4.0	7.225	1.280	20.296
		4.2	7.243	1.268	20.249
		Mean ± SD	7.257 ± 0.016	1.272 ± 0.006	20.272 ± 0.023
		%RSD	0.225	0.504	0.115
PCM	Mobile phase ratio	Buffer 92%	3.379	1.350	12.368
		Buffer 93%	3.413	1.352	12.290
		Buffer 94%	3.381	1.354	12.375
		Mean ± SD	3.391 ± 0.019	1.352 ± 0.002	12.344 ± 0.047
		%RSD	0.562	0.147	0.382
	Flow Rate	1.9	3.403	1.302	12.310
		2.0	3.413	1.352	12.290
		2.1	3.392	1.329	11.899
		Mean ± SD	3.402 ± 0.010	1.327 ± 0.025	12.166 ± 0.231
		%RSD	0.308	1.885	1.904
	pH	3.8	3.392	1.324	12.160
		4.0	3.413	1.352	12.290
		4.2	3.318	1.338	11.887
		Mean ± SD	3.374 ± 0.049	1.338 ± 0.014	12.112 ± 0.205
		%RSD	1.478	1.046	1.698
PAP	Mobile phase ratio	Buffer 92%	1.742	1.551	
		Buffer 93%	1.728	1.550	–
		Buffer 94%	1.779	1.552	–
		Mean ± SD	1.749 ± 0.026	1.551 ± 0.001	
		%RSD	1.506	0.064	
	Flow Rate	1.9	1.759	1.54	–
		2.0	1.728	1.55	–
		2.1	1.700	1.497	–
		Mean ± SD	1.729 ± 0.029	1.529 ± 0.028	
		%RSD	1.706	1.841	
	pH	3.8	1.789	1.540	–
		4.0	1.728	1.550	–
		4.2	1.789	1.590	–
		Mean ± SD	1.768 ± 0.035	1.560 ± 0.026	
		%RSD	1.991	1.695	
PHA	Mobile phase ratio	Buffer 92%	4.979	1.459	14.376
		Buffer 93%	5.003	1.485	14.297
		Buffer 94%	4.971	1.450	14.498
		Mean ± SD	4.95 ± 0.068	1.46 ± 0.018	14.39 ± 0.101
		%RSD	1.372	1.240	0.703
	Flow Rate	1.9	4.992	1.362	14.111
		2.0	5.003	1.352	14.498
		2.1	4.992	1.377	14.373
		Mean ± SD	4.99 ± 0.006	1.36 ± 0.012	14.32 ± 0.197
		%RSD	0.127	0.922	1.378
	pH	3.8	4.992	1.369	14.461
		4.0	5.003	1.352	14.498
		4.2	4.981	1.399	14.278
		Mean ± SD	4.99 ± 0.011	1.37 ± 0.023	14.41 ± 0.117
		%RSD	0.220	1.732	0.817

### Method validation

The proposed method was validated in accordance to ICH guidelines [56], as shown in Table 4.

**Table 4:** Assay validation of the proposed method for the determination of ETO, PCM, PAP, and PHA as per ICH guidelines

Parameters	Proposed HPLC method			
	ETO	PCM	PAP	PHA
Range (μg/mL)	1.5–30	1.5–30	0.5–10	0.5–10
Linearity				
Slope	263,046	189,792	204,882	231,325
Intercept	50,255	934,351	112,694	25,478
Correlation coefficient (r)	0.9999	0.9993	0.9996	0.9998
Accuracy ^a^ (Mean ± %SD)	100.21 ± 0.973	100.58 ± 0.858	98.17 ± 1.362	99.32 ± 1.163
Precision (%RSD)				
Repeatability ^b^	0.942	1.388	1.361	1.270
Intermediate ^c^	1.344	1.422	1.588	1.454
LOD (μg/mL) ^d^	0.304	0.397	0.113	0.062
LOQ (μg/mL) ^d^	0.923	1.204	0.343	0.189

^a^ Accuracy (mean ± %RSD) [average of three different concentrations of three replicates each (n = 9)]

^b^ The intraday (n = 9), RSD % of three concentrations (10,15, 30 μg/mL) for ETO, PCM, and (3,5,10 μg/mL) for PAP, and PHA, respectively; repeated three times within the same day

^c^ The interday (n = 9), RSD % of three concentrations (10,15, 30 μg/mL) for ETO, PCM, and (3,5,10 μg/mL) for PAP, and PHA, respectively; repeated three times in three successive days

^d^ LOD and LOQ are calculated using the formula LOD = 3.3*(SD of intercept /slope) and, LOQ = 10*(SD of intercept /slope), respectively

**Fig. 3:**
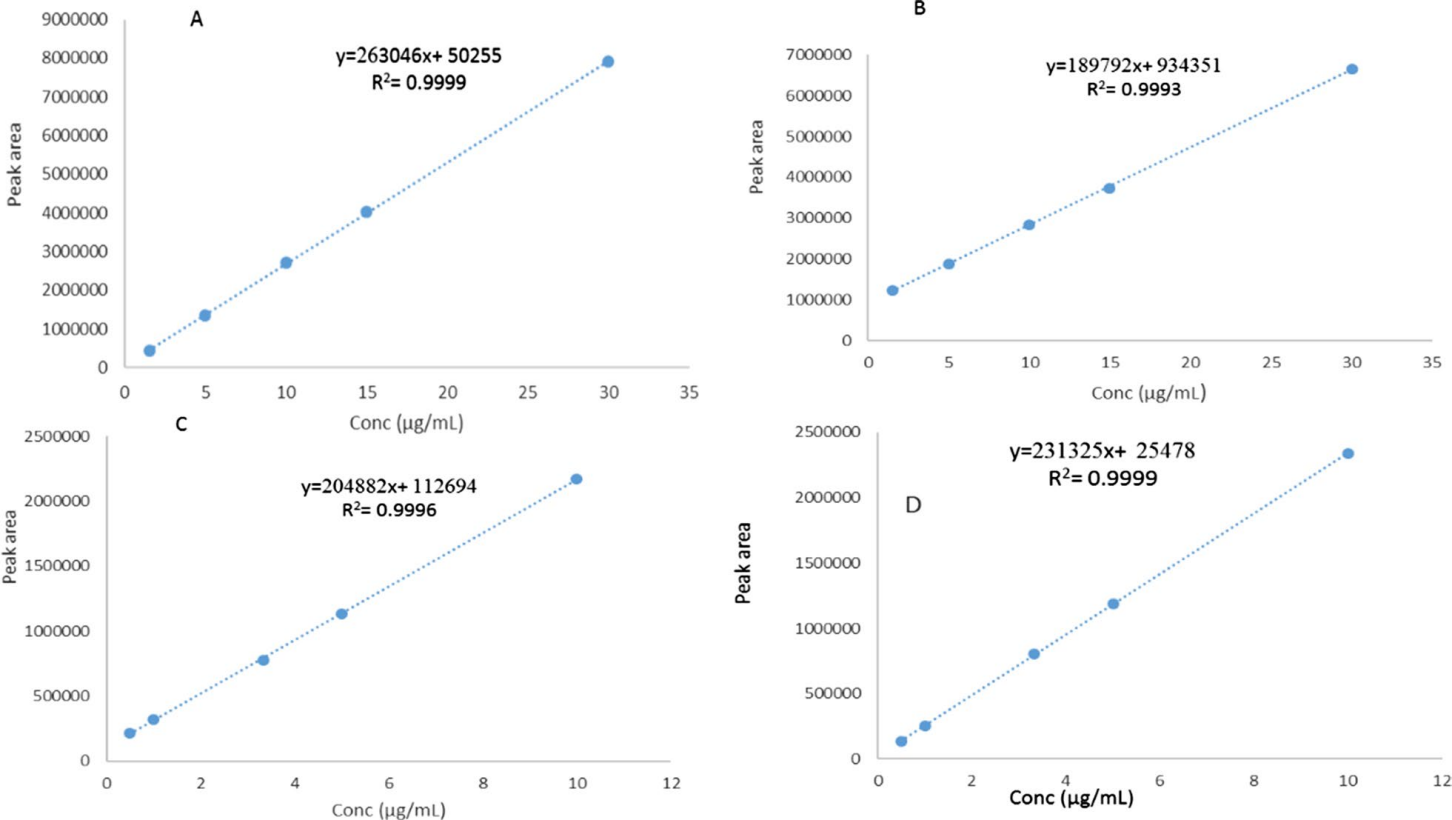
Calibration curve of **a** Etoricoxib, **b** paracetamol, **c** 4-aminophenol and **d** para-hydroxy acetophenone

The suggested method's calibration curves were generated to illustrate the relationship between the mean peak area and the corresponding concentration in range of 1.5–30 µg/mL for ETO and PCM and 0.5–10 µg/mL for PAP and PHA, as shown in Fig. 3. The regression equations have been computed and results are shown in Table 4.

The accuracy of the proposed method was assessed across the required range by analyzing three different concentrations of pure samples in triplicate, and the mean of the percentage recoveries ± SD was calculated (100.21 ± 0.973, 100.58 ± 0.858, 98.17 ± 1.362, and 99.32 ± 1.163) for ETO, PCM, PAP, and PHA, respectively, confirming accuracy of the method as shown in Table 4.

Repeatability and intermediate precision were also investigated, three different concentration levels within the specified range, either within the same day or on three successive days to investigate intra-day and inter-day precision, respectively. As proven by the low %RSD values, the suggested analytical procedure yielded data with acceptable precision (Table 4).

Limit of detection (LOD) and limit of quantitation (LOQ) were calculated for ETO, PCM, PAP, and PHA based on the standard deviation of the intercept (SD) and the slope obtained from the calibration curves of each component. The low LOD values (0.304, 0.397, 0.113, and 0.062) were obtained, demonstrating the proposed method's great sensitivity for ETO, PCM, PAP, and PHA, respectively, as shown in Table 4.

### Assay of pharmaceuticals dosage form

**Fig. 4:**
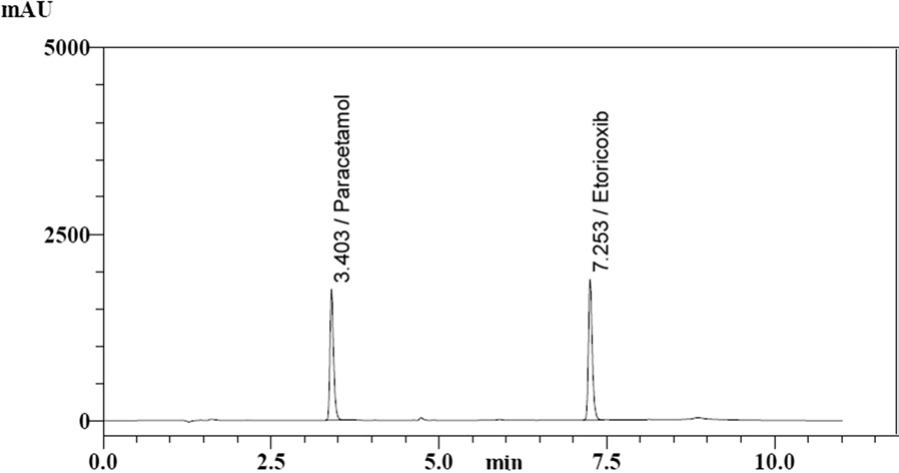
HPLC chromatogram of sample solution (dosage form)

The proposed method successfully determined ETO and PCM in Intacoxia-P tablets. The standard addition technique was employed to validate the proposed method for determining ETO and PCM selectively in the presence of formulation excipients and additives, and good results were obtained (Table 5; Fig. 4).

**Table 5:** Determination of ETO and PCM in pharmaceutical formulation and application of the standard addition technique using the proposed HPLC method

Pharmaceutical formulation	Drug	Found% ± SD ^a^	Standard addition			
			Claimed taken (µg/mL)	Pure added (µg/mL)	Found (µg/mL)	Recovery% ^b^
Intacoxia-P tablet (each tablet labeled to contain 60 mg ETO and 325 mg PCM)	ETO	99.50 ± 1.207	1.8	1.8	1.76	98.10
				3	2.98	99.46
				4.5	4.48	99.76
	Mean ± SD					99.11 ± 0.844
	%RSD					0.893
	PCM	100.22 ± 0.948	10	2	1.97	98.95
				5	4.96	99.36
				10	10.03	100.31
	Mean ± SD					99.54 ± 0.697
	%RSD					0.701

^a^ Application (mean ± SD) [average of three determinations of 1.8 µg/mL for ETO, and 10 µg/mL for PCM)

^b^ Average of three determinations

### Statistical comparison

Results obtained from the analysis of pure ETO and PCM were statistically compared with those obtained by the reported HPLC method [49]. The comparison revealed that the calculated F and student’s t-test values are less than the tabulated ones, revealing no significant difference between the proposed and reported method as shown in Table 6.

**Table 6:** Statistical comparison of the results obtained by the proposed HPLC method and reported method for the determination of ETO and PCM

Parameter	HPLC method		Reported HPLC method ^a^	
	ETO	PCM	ETO	PCM
Mean	100.21	100.58	99.98	99.89
SD	0.973	0.858	0.992	1.102
Variance	0.947	0.736	0.984	1.214
n	9	9	9	9
Student's t-test ^b^ (2.120)	0.492	1.479		
F value ^b^ (3.44)	1.040	1.650		

^a^ HPLC method using a C18 column as the stationary phase and a mixture consisting of methanol: water) in ratio (70:30 v/v as a mobile phase. The mobile phase was pumped at a flowrate of 1.0 mL/min. UV detection was carried out at 235.0 nm [49]

^b^ The values in parentheses are the corresponding tabulated two-tailed values at _*P*_ = 0.05

The proposed method was compared to the other published methods [49–53] and the findings showed that the proposed method was more sensitive than the published ones. The proposed method excels the published ones as it separates and quantifies PCM toxic impurities as well as PCM and ETO mixtures, which was not published before, as shown in Table 7.

**Table 7:** Comparison of the proposed HPLC method to the reported HPLC methods

Reported methods	Analytes separated		Linearity (µg/mL) of the drugs	Retention time
	Impurities	Drugs		
Ref (49)	–	PCM	5–30	3.07
		ETO	5–30	5.72
Ref (50)	–	PCM	50–150	3.27
		ETO	6–18	6.12
Ref (51)	–	PCM	8.3–41.5	3.12
		ETO	1–5	6.82
Ref (52)	–	PCM	48–146	8.34
		ETO	6 -19	18.45
Ref (53)	–	PCM	1200–3600	2.460
		ETO	1000 -3000	1.189
Proposed method	PAP, PHA	PCM	1.5–30 μg /mL	3.41
		ETO	1.5–30 μg /mL	7.27

## Conclusion

A validated, robust, precise, accurate, and selective gradient RP-HPLC method was used to determine PCM and ETO in pharmaceutical preparations without interference from PCM impurities. The methodology was validated in agreement with the ICH recommendations. The results show that using a linear gradient system with respect to the mobile phase allows the separation of studied drugs and impurities with high resolution and relatively short analysis time. The proposed method was shown to be suitable for use in quality control laboratories for determining PCM in pure form or pharmaceutical dosage forms with ETO.

## Data Availability

Datasets generated and/or analyzed during the current study are available from the corresponding author on reasonable request.
